# Protofibril–Fibril Interactions Inhibit Amyloid Fibril Assembly by Obstructing Secondary Nucleation

**DOI:** 10.1002/anie.202010098

**Published:** 2020-12-11

**Authors:** Filip Hasecke, Chamani Niyangoda, Gustavo Borjas, Jianjun Pan, Garrett Matthews, Martin Muschol, Wolfgang Hoyer

**Affiliations:** ^1^ Institut für Physikalische Biologie Heinrich-Heine-Universität Düsseldorf 40204 Düsseldorf Germany; ^2^ Strukturbiochemie (IBI-7) Forschungszentrum Jülich 52425 Jülich Germany; ^3^ Department of Physics University of South Florida Tampa FL 33620 USA

**Keywords:** aggregates, fibrils, peptides, protein–protein interactions, self-assembly

## Abstract

Amyloid‐β peptides (Aβ) assemble into both rigid amyloid fibrils and metastable oligomers termed AβO or protofibrils. In Alzheimer's disease, Aβ fibrils constitute the core of senile plaques, but Aβ protofibrils may represent the main toxic species. Aβ protofibrils accumulate at the exterior of senile plaques, yet the protofibril–fibril interplay is not well understood. Applying chemical kinetics and atomic force microscopy to the assembly of Aβ and lysozyme, protofibrils are observed to bind to the lateral surfaces of amyloid fibrils. When utilizing Aβ variants with different critical oligomer concentrations, the interaction inhibits the autocatalytic proliferation of amyloid fibrils by secondary nucleation on the fibril surface. Thus, metastable oligomers antagonize their replacement by amyloid fibrils both by competing for monomers and blocking secondary nucleation sites. The protofibril—fibril interaction governs their temporal evolution and potential to exert specific toxic activities.

## Graphical abstract

Amyloid fibrils preferentially originate from the surface of existing fibrils, as the fibril surface catalyzes the conversion of monomeric precursors in a process called secondary nucleation. Metastable protofibrils, an alternative, particularly toxic, assembly type of amyloid proteins, bind amyloid fibril surfaces and obstruct secondary nucleation. Protofibrils thereby delay their own replacement by amyloid fibrils.

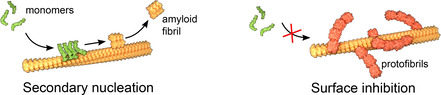

## Introduction

Amyloid fibrils are cross‐β structured protein assemblies that represent the hallmark of many protein aggregation disorders.^[1]^ For several disease‐related proteins, amyloid fibrils correspond to the thermodynamic minimum of the free energy landscape for folding and aggregation.^[2]^ For example, Aβ amyloid fibrils are the core components of the senile plaques found in Alzheimer's disease (AD)‐affected brains.^[3]^ Aβ fibrils are polymorphic, variably constructed from in‐register parallel β‐sheets.[^4^, ^5^, ^6^] They form by nucleated polymerization, where initial fibril nuclei grow by monomer addition to the fibril ends.^[7]^ A frequent contributor to the typical sigmoidal growth profile of amyloid fibrils is fibril‐mediated secondary nucleation. In this process, the fibril surface acts as the preferential site for new fibril nucleation, leading to the autocatalytic proliferation of amyloid fibrils.^[7]^

A second type of assemblies that Aβ is prone to form are metastable globular oligomers with a molecular weight >50 kD, and their associated curvilinear fibrils with typical lengths up to 200 nm.[^8^, ^9^, ^10^, ^11^, ^12^, ^13^, ^14^] These oligomers are collectively referred to as AβO or protofibrils.[^8^, ^12^, ^15^] As these oligomers are formed in a reaction distinct from fibril formation (i.e., off‐pathway),[^8^, ^11^, ^13^, ^16^] the term protofibril can be misleading. Similarly, the term AβO is used interchangeably for on‐pathway oligomers. Below we use the designations globular oligomer (gO) and curvilinear fibril (CF) to refer specifically to the off‐pathway, metastable assemblies. GO/CFs form in a lag‐free oligomerization reaction with a much higher reaction order than that observed for fibril formation.^[11]^ Like amyloid fibrils, gO/CFs are rich in β‐sheets, but their structure has not been resolved to the same level of detail yet.^[17]^ GO/CFs have been reported for several amyloidogenic proteins, suggesting that they are a general alternative assembly type of this class of proteins.[^16^, ^18^, ^19^, ^20^]

Aβ gO/CFs may represent the main toxic species in AD, as they are more effective than amyloid fibrils at inducing synaptic dysfunction, inhibiting long‐term potentiation, triggering inflammation, and disrupting membranes.[^8^, ^13^] Several receptors that mediate toxic signaling of extracellular Aβ gO/CFs have been identified.^[21]^ In addition, intracellular Aβ gO/CFs show cytotoxic effects.^[8]^ Aβ gO/CFs are enriched in a halo surrounding senile plaques, pointing to a potential role of gO/CF‐fibril interactions.[^22^, ^23^] For example, fibril plaques have been suggested to serve as a reservoir, or buffer, of Aβ oligomers.[^22^, ^23^] However, gO/CF–fibril interactions have not been characterized in detail.

We have recently reported that the high concentration dependence of gO/CF formation results in a threshold monomer concentration required for gO/CF formation, denoted critical oligomer concentration (COC), which is significantly higher than the threshold for fibril formation.[^11^, ^20^] Above the COC, the assembly kinetics are biphasic, with an initial lag‐free gO/CF formation phase, followed by a sigmoidal phase representing the nucleation and growth of fibrils which slowly replace the metastable gO/CFs. Surprisingly, we observed that gO/CF formation above the COC progressively increased the lag period for subsequent fibril nucleation and growth, revealing that gO/CFs inhibit fibril formation not only by competing for monomers, but also in an active fashion. These observations were made with two distinct amyloid proteins, a dimeric variant of Aβ40 (dimAβ) and hen egg‐white lysozyme (hewL).^[11]^

Here, we investigate how gO/CFs actively inhibit fibril formation. We first show that the inhibitory effects of off‐pathway gO/CF formation on subsequent fibril nucleation and growth are similarly present in the two dominant AD peptides Aβ40 and Aβ42. We then demonstrate for Aβ as well as for hewL that gO/CFs bind to fibril surfaces. GO/CF binding also promotes fibril bundling, thereby further reducing fibril surface area. We finally take advantage of the Aβ‐dimAβ system to show that the gO/CF‐fibril interaction interferes with secondary nucleation and blocks the proliferation of amyloid fibrils.

## Results and Discussion

**Figure 1:**
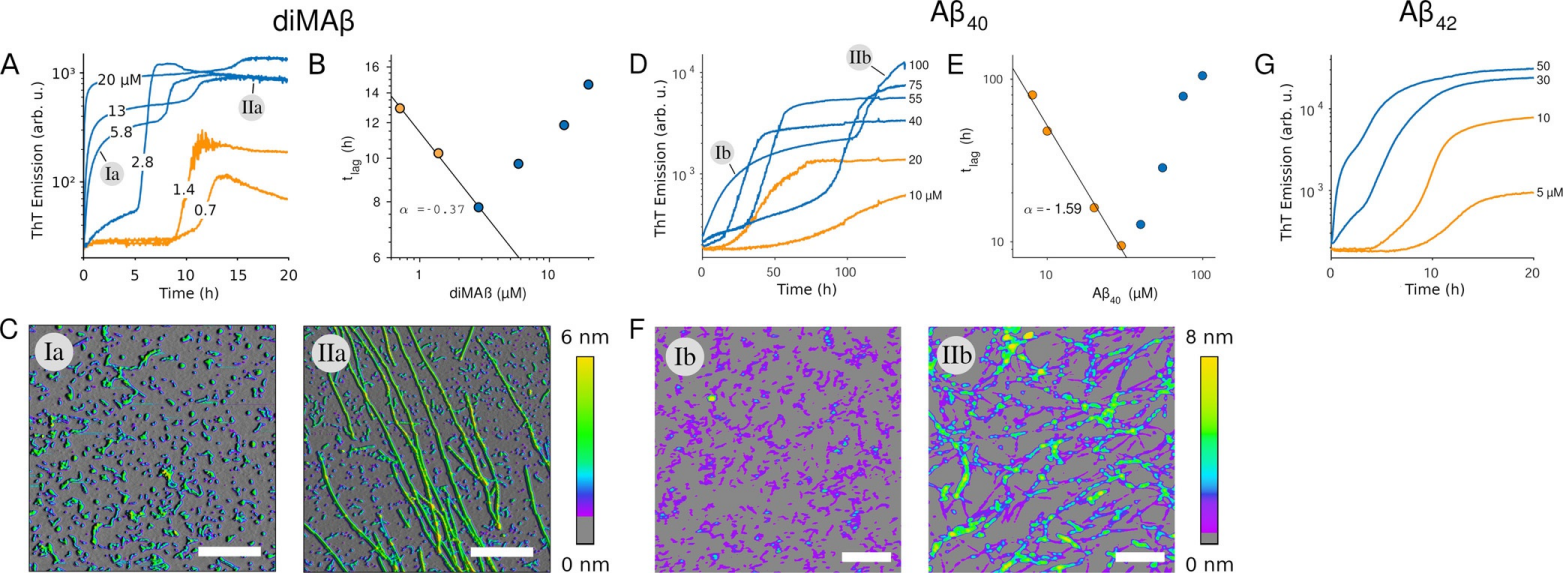
Biphasic assembly kinetics of Aβ. A), D), G) Transition from sigmoidal (orange) to bimodal (blue) amyloid growth kinetics of dimAβ, Aβ40, and Aβ42, monitored by ThT fluorescence. Concentration dependent time traces of A) dimAβ assembly in 50 mM Na‐phosphate, 50 mM NaCl, pH 7.4, 37 °C, and D) Aβ40 or G) Aβ42 assembly in 50 mM Na‐phosphate, pH 7.4, 27 °C. ThT fluorescence is plotted logarithmically to highlight the stable low signal during the lag‐time under sigmoidal growth conditions. B), E) Dependence of the lag‐time of the second kinetic phase on protein concentration. C), F) AFM images corresponding to the early oligomeric and subsequent fibril‐dominated kinetic phases observed above the COC.

To investigate gO/CF formation of Aβ, we have generated dimAβ, a dimeric Aβ variant in which two Aβ40 units are linked in one polypeptide chain through a flexible glycerin‐serine‐rich linker.^[11]^ The conformational properties of the Aβ40 units in dimAβ are the same as those of unlinked Aβ40.^[11]^ However, due to the increased local Aβ concentration, gO/CF formation of dimAβ is strongly promoted, which is reflected in the comparatively low COC of ≈1.5 μM at neutral pH.^[11]^ Above the COC, Thioflavin T (ThT) fluorescence indicates biphasic assembly kinetics of dimAβ (Figure 1 A). During the first phase, gO/CFs form (Figure 1 C) in an oligomerization reaction with a high reaction order of ≈3.^[11]^ After a lag‐time, amyloid fibril formation is observed, in agreement with a nucleation‐polymerization reaction (Figure 1 A,C).^[11]^ Upon prolonged incubation, the metastable gO/CFs are slowly replaced by amyloid fibrils.^[11]^ Above the COC, the lag‐time of amyloid fibril formation develops an inverse dependence on protein concentration, i.e., the lag‐time increases with protein concentration (Figure 1 B), indicating that gO/CFs actively interfere with amyloid fibril formation.^[11]^

We tested if these observations, previously made for dimAβ and hewL, are reproduced for Aβ40 and Aβ42. A logarithmic plot of the ThT time course of Aβ40 assembly at a concentration of 20 μM or below shows a sigmoidal curve with a lag‐time of several hours. This is in agreement with amyloid formation by a nucleation‐polymerization reaction with prominent contributions from secondary nucleation (Figure 1 D). In contrast, for Aβ40 concentrations of 40 μM or above, an additional, lag‐free kinetic phase occurred during which gO/CFs assembled (Figure 1 D,F). These gO/CFs were replaced by amyloid fibrils during a second kinetic phase (Figure 1 D,F). Aβ40 assembly thus follows the same pattern as dimAβ assembly, albeit with an approximately 20‐fold higher COC (≈30 μM), which is expected considering the lack of a covalent connection between Aβ monomers in unlinked Aβ40. ThT kinetics recorded with Aβ40 by the deGrado and Prusiner lab, for concentrations at or above those used here, generated similar biphasic kinetics and produced long‐lived Aβ gOs.^[24]^ As with dimAβ and hewL, the lag‐time of amyloid fibril formation of Aβ40 started to increase above the COC (Figure 1 E). This indicates that Aβ40 gO/CFs share the ability to interfere actively with fibril formation. For Aβ42, the ThT time courses indicated a transition to biphasic kinetics at a concentration between 10 and 30 μM (Figure 1 G), in line with previous observations.^[25]^ The short lag times of Aβ42 amyloid fibril formation undermined our efforts of correlating biphasic ThT kinetics with the onset of gO/CF formation in that system. Nevertheless, the data for Aβ40 and Aβ42 show that the observations made for dimAβ and hewL extend to the two prevalent Aβ variants, with higher COCs of the unlinked peptides.

**Figure 2:**
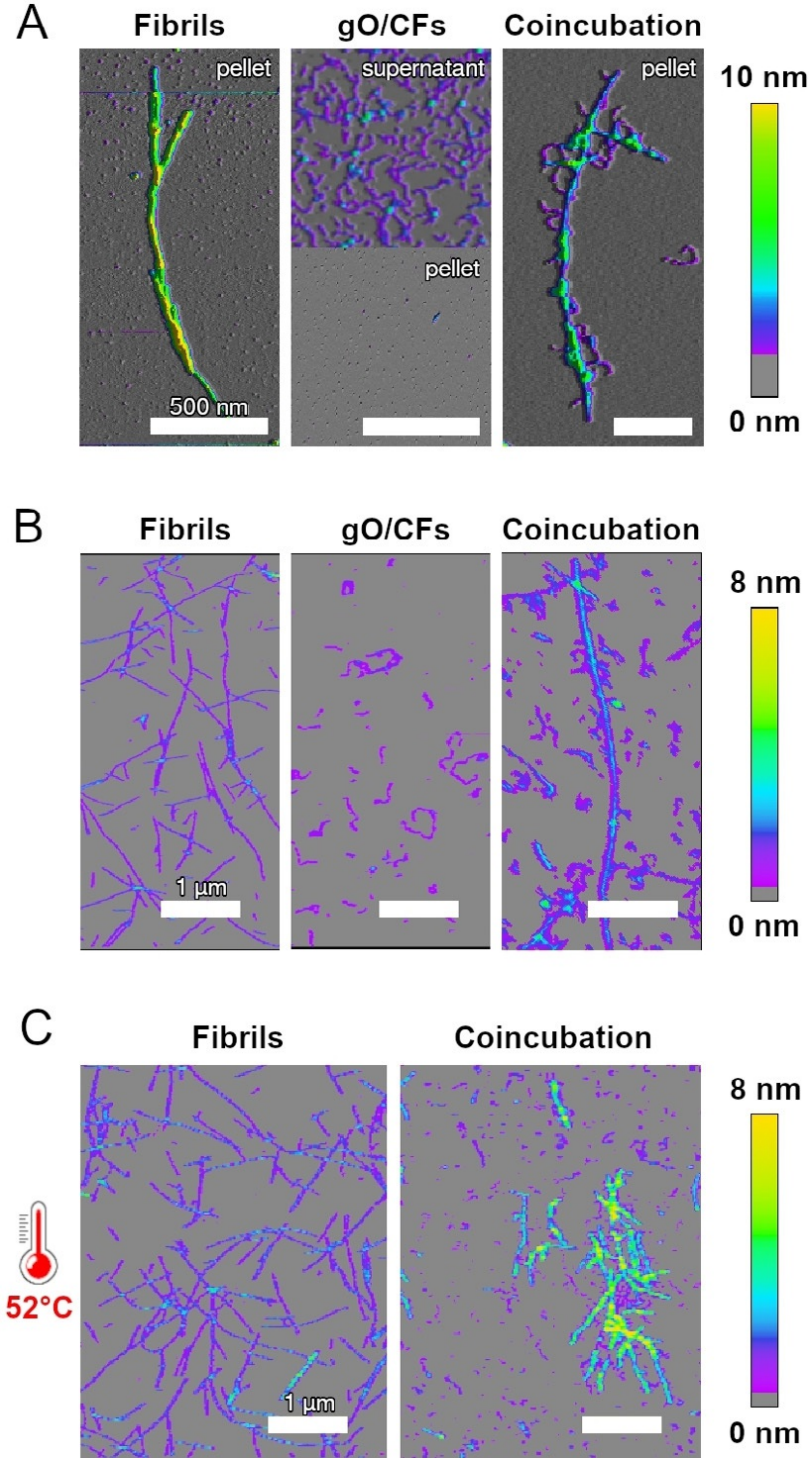
GO/CFs bind to amyloid fibril surfaces. AFM images of assemblies of A) dimAβ and Aβ40 or B),C) hewL. A) Amyloid fibrils formed from 10 μM Aβ40 were found in the pellet upon centrifugation at 14 000 g (left); gO/CFs formed from 10 μM dimAβ remained in the supernatant (middle). Upon mixing equimolar amounts, dimAβ gO/CFs co‐precipitated with Aβ40 fibrils and decorated fibril surfaces (right). B) Amyloid fibrils and gO/CFs formed from 1.75 mM hewL were grown below (50 mM NaCl) or above (250 mM NaCl) the COC, respectively. After isolation and adjusting NaCl for both to 450 mM,100 μM of fibrils were mixed with 1 mM of gO/CFs at room temperature and in 450 mM NaCl. C) Mixing hewL gO/CFs and fibrils at growth temperature (52 °C), instead, induced rapid fibril bundling and precipitation while, under the same conditions, fibrils themselves remained unchanged.

One possible mechanism by which gO/CFs might actively inhibit amyloid formation would be by interfering with secondary nucleation. GO/CFs might bind to amyloid fibril surfaces, where they could block the sites capable of catalyzing fibril nucleation. To test this hypothesis, we first investigated if gO/CFs bind to amyloid fibril surfaces. Fibrils were formed from Aβ40 at a concentration of 10 μM. Since this concentration is below the COC of Aβ40, only fibrils but no gO/CFs were formed. Upon centrifugation, the fibrils were found in the pellet (Figure 2 A, left). GO/CFs were formed by quiescently incubating dimAβ at a concentration of 10 μM for 24 hours. Under these conditions dimAβ assembled into gO/CFs whereas amyloid fibrils were still absent. The gO/CFs were collected from the supernatant after centrifugation (Figure 2 A, middle). When Aβ40 fibrils and dimAβ gO/CFs were mixed and subsequently centrifuged, the pellet contained amyloid fibrils decorated with gO/CFs (Figure 2 A, right). This indicates that the fibril surfaces have an affinity for gO/CFs, leading to co‐precipitation of the two species. The experiment was repeated for hewL. HewL amyloid fibrils grown under sigmoidal (sub‐COC) conditions (Figure 2 B, left) and hewL gO/CFs formed during the early phases of biphasic growth (Figure 2 B, middle) were mixed, resulting in binding of gO/CFs to the lateral surfaces of the fibrils (Figure 2 B, right). In addition, mixing of hewL gO/CFs with fibrils at growth temperatures dramatically increased lateral bundling and precipitation of fibrils, while isolated fibrils remained unchanged (Figure 2 C). Both binding and bundling reduce the fibril surface area available for secondary nucleation.

**Figure 3:**
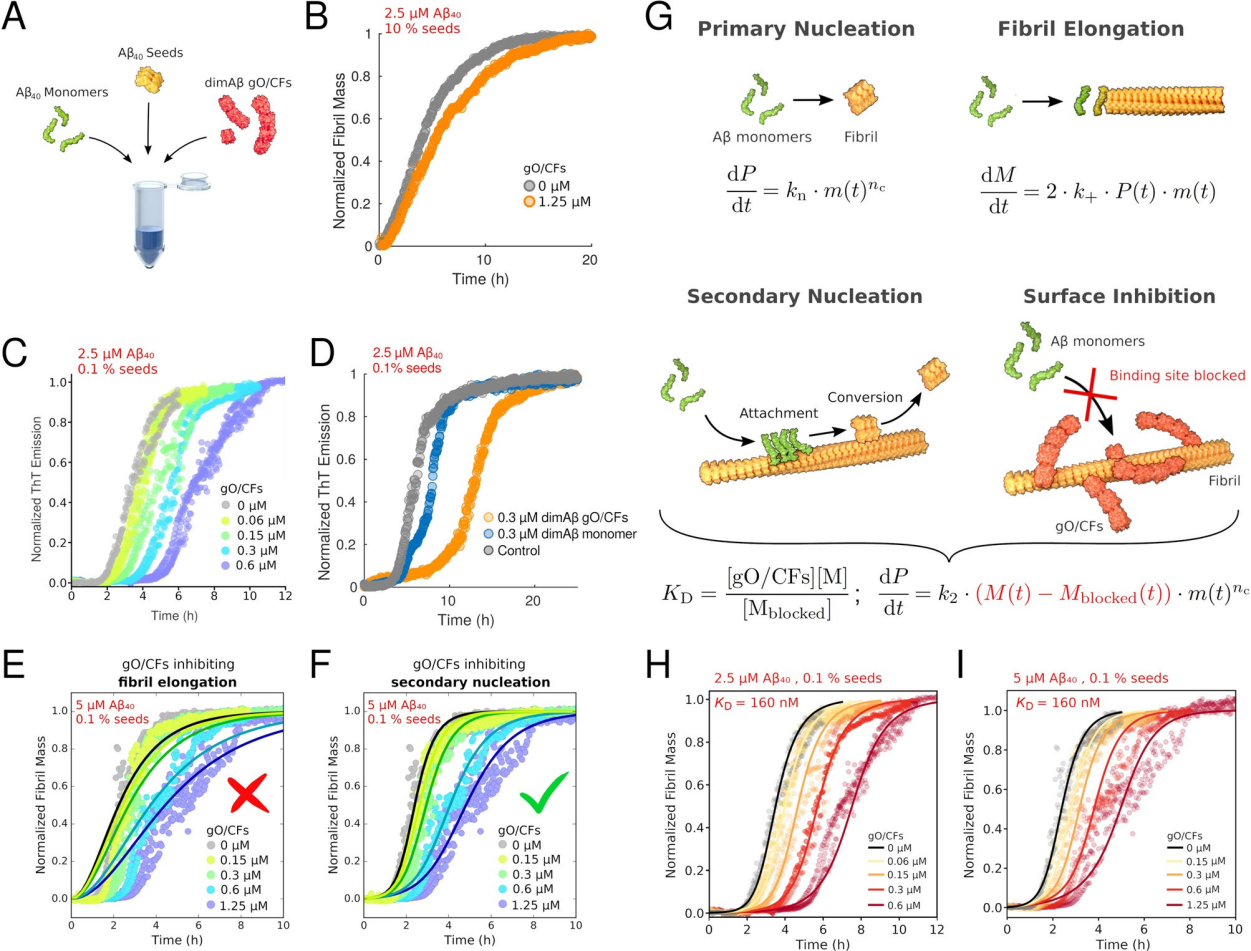
GO/CFs inhibit secondary nucleation of amyloid fibrils. A) Scheme of the kinetics assays. The effects of dimAβ gO/CFs on secondary nucleation and elongation of Aβ40 amyloid fibrils were probed. B) Elongation of Aβ40 fibril seeds by Aβ40 monomers in the absence and presence of dimAβ gO/CFs. C) Secondary nucleation‐elongation of Aβ40 fibril seeds by Aβ40 monomers in the absence and presence of dimAβ gO/CFs. D) Secondary nucleation‐elongation of Aβ40 fibril seeds by Aβ40 monomers in the absence (grey) and presence of dimAβ gO/CFs formed above the COC and diluted below the COC (orange) or dimAβ monomers below the COC (blue). E) Global fits to the data using a nucleation‐elongation model. All parameters were shared apart from the elongation rate constants. F) Global fits to the data using a secondary nucleation‐elongation model. All parameters were shared apart from the secondary nucleation rate constant. G) Nucleation‐growth model including binding of gO/CFs to amyloid fibril surfaces, which inhibits secondary nucleation. P, fibril particle concentration; M, fibril mass concentration; m, monomer concentration; *n*_c_, nucleus size; *k*_n_, primary nucleation rate constant; *k*_2_, secondary nucleation rate constant; *k*_+_, elongation rate constant; *K*_D_, affinity of gO/CF for the fibril surface. H), I) Numerical simulations applying the model outlined in G), using the rate constants obtained for the nucleation‐elongation model in F) (uninhibited trace) and a *K*_D_ of 160 nM. Duplicate or triplicate measurements per condition are shown in panels (C), (E), (F), (H), (I).

In order to isolate the consequences of this gO/CF and fibril interaction on fibril growth mechanisms we performed seeded fibril growth experiments with increasing gO/CF admixtures. To do so, we took advantage of the different COCs for dimAβ vs. Aβ40: at low μM concentrations dimAβ assembles into gO/CFs, whereas Aβ40 continues to exhibit the sigmoidal kinetics of nucleated‐polymerization with secondary nucleation. Furthermore, dimAβ gO/CFs possess high kinetic stability and persist even for several hours after dilution to sub‐COC concentrations, thereby allowing to investigate effects of gO/CFs down to sub‐μM concentrations.^[26]^ Amyloid fibril formation is a multistep reaction (Figure 3 G).^[27]^ To test the effects of gO/CFs specifically on fibril elongation and secondary nucleation, we seeded Aβ40 monomers with different concentrations of sonicated Aβ40 fibrils in the presence of increasing concentrations of dimAβ gO/CFs (Figure 3 A). When 10 % Aβ40 seeds were added to 2.5 μM Aβ40 monomers, fibril elongation was the dominant reaction as evident from the immediate linear increase in ThT signal (Figure 3 B). Addition of 1.25 μM dimAβ gO/CFs (corresponding to an Aβ40 subunit concentration of 2.5 μM) did not have a substantial effect, showing that gO/CFs do not actively interfere with amyloid fibril elongation (Figure 3 B). When a lower amount, that is, 0.1 %, of Aβ40 seeds was applied, sigmoidal time traces were obtained, indicating the importance of autocatalytic amplification of amyloid fibrils by secondary nucleation (Figure 3 C). In this case, addition of dimAβ gO/CFs led to a concentration‐dependent increase in lag‐time (Figure 3 C). Since primary nucleation does not contribute to the ThT signal on this time scale at this Aβ40 monomer concentration (Figure 1 D) and fibril elongation is not affected by gO/CFs (Figure 3 B), we conclude that gO/CFs inhibit secondary nucleation. The inhibitory effect was already discernible at a concentration of 60 nM gO/CFs, which corresponds to a gO/CF:monomer ratio of 1:20 in numbers of Aβ40 units. Such a substoichiometric effect is compatible with inhibition of an autocatalytic process. To confirm that inhibition of Aβ40 fibril formation is in fact caused by gO/CFs and not due to any other activity of dimAβ on Aβ40, we compared the effects of i) dimAβ gO/CFs prepared above the COC and diluted to a sub‐COC concentration of 0.3 μM with those of ii) dimAβ monomers that were freshly eluted from size exclusion chromatography and kept at a sub‐COC concentration of 0.3 μM. The dimAβ preparation that contained gO/CFs due to incubation above the COC exhibited a much stronger effect on fibril formation than the one kept below the COC (Figure 3 D). The inhibition is not an unspecific effect of any polypeptide assembly in the size range of gO/CFs, as it is not observed for ferritin, a 24‐mer of helical bundles with a molecular weight of 440 kD (Figure S1).

To further confirm that the kinetics data are in agreement with inhibition of secondary nucleation, we computed global fits to the gO/CF concentration‐dependent data for two different models of fibril formation using the software package Amylofit.^[27]^ First, we applied a nucleation‐elongation model and performed global fits that attributed the effects of gO/CFs to an altered rate constant of either primary nucleation or fibril elongation (all parameters were shared among the data sets apart from the rate constants of primary nucleation or fibril elongation, respectively). These fits showed clear deviations from the experimental data (Figures 3 E and S2A,B). Second, we applied a secondary nucleation‐elongation model and performed global fits that attributed the effects of GO/CFs to altered rate constants of either primary nucleation, secondary nucleation, or fibril elongation (again, keeping all other fitting parameters the same among the data sets). The global fit to this model using a variable rate constant of primary nucleation did not reproduce the decreasing slope during the exponential growth phase with increasing gO/CF concentration (Figure S2C). In contrast, when the rate constants of secondary nucleation or fibril elongation were variable, good agreement with the data was obtained (Figures 3 F and S2D,E). These fits do not differentiate between effects on secondary nucleation and fibril elongation, as the rate constant of secondary nucleation occurs in the regression equation only in the form of its product with the rate constant of fibril elongation.^[28]^ However, as we can exclude any substantial effect of gO/CF on fibril elongation (Figure 3 B), the global fits further strengthen the case for gO/CFs inhibiting amyloid fibril formation through an effect on secondary nucleation. As gO/CFs bind to amyloid fibril surfaces, they likely inhibit secondary nucleation by blocking the sites capable of catalyzing secondary nucleation (Figure 3 G). This mode of inhibition of Aβ fibril formation has previously been described for the BRICHOS chaperone.^[29]^ The reduction in the number of active sites effectively corresponds to a reduction in the fibril surface available for autocatalytic amplification rather than to a decrease in the secondary nucleation rate constant. We extended the nucleation‐polymerization model by including an equilibrium of gO/CF binding to fibrils that reduces the fibril mass engaged in secondary nucleation (Figure 3 G). Numerical simulations with the modified model were performed, using the rate constants obtained by Amylofit for the uninhibited case of nucleation‐polymerization with variable secondary nucleation (black fit in Figure 3 F). In particular, the same secondary nucleation rate constant was used for all gO/CF concentrations, attributing the gO/CF concentration dependence of the kinetics solely to changes in the fibril mass available for secondary nucleation according to the gO/CF:fibril interaction equilibrium. The gO/CF:fibril interaction was treated as a 1:1 interaction in the number of Aβ subunits. When applying a dissociation constant of *K*_D_=160 nM the numerical simulations yielded good agreement with data obtained both at 2.5 μM and 5 μM Aβ40 monomer concentration (Figure 3 H,I).

## Conclusion

We previously observed a remarkable inversion of the scaling relation between increasing protein concentration and decreasing lag‐times for dimAβ and hewL amyloid fibril formation upon crossing the COC.^[11]^ Here, we reproduced the surprising increase in lag‐time with increasing protein concentration for Aβ40, which indicates that gO/CFs actively inhibit fibril formation (Figure 1 E). Collectively, the AFM data (Figure 2) and chemical kinetics data (Figure 3) provide strong evidence that gO/CFs inhibit Aβ amyloid fibril formation by binding to amyloid fibril surfaces, blocking the sites that would otherwise promote secondary nucleation. The same mode of inhibition was observed for the BRICHOS chaperone, but not for a set of control proteins.^[29]^ This suggests that this inhibitory activity is rather specific. It is also in line with the relatively high affinity of the gO/CF:fibril interaction, as indicated by the observed inhibition at low nM gO/CF concentration.

Our observations provide insight into the structure specificity of secondary nucleation. Decoration of amyloid fibril surfaces with gO/CFs formed from the same protein results in less efficient secondary nucleation. This demonstrates that gO/CF surfaces do not possess the same capacity as amyloid fibril surfaces to catalyze fibril nucleation, suggesting that the cross‐β structure of amyloid fibrils is essential for efficient secondary nucleation. This is consistent with the distinct structural signatures of gO/CFs vs. fibrils seen in the amide‐I bands of their respective infrared spectra that we have shown for hewL and that have been reported for Aβ, as well.[^20^, ^30^]

**Figure 4:**
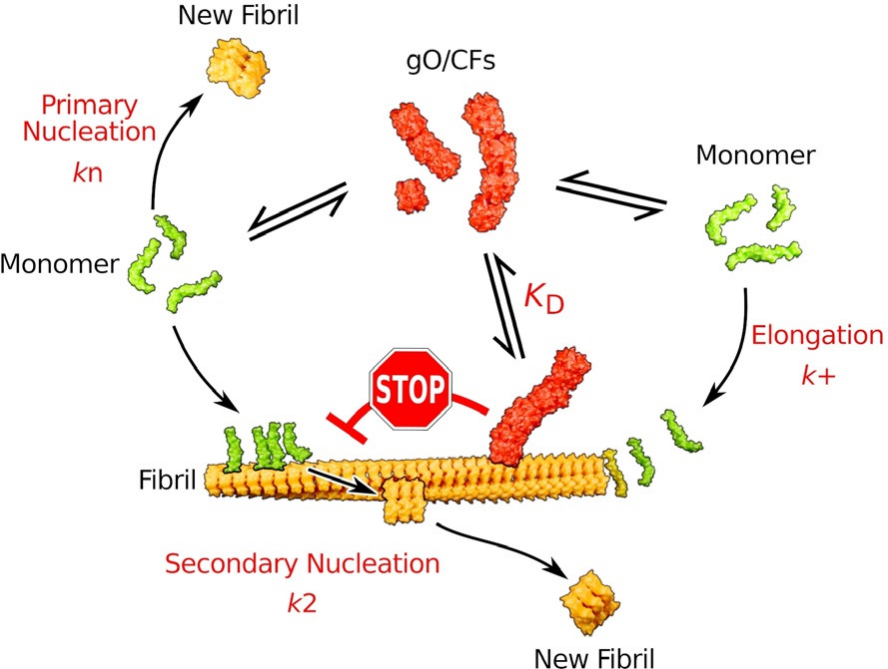
Scheme of oligomer and amyloid fibril formation. GO/CFs constitute an alternative (off‐pathway) assembly type that competes with amyloid fibrils for monomers and that inhibits the autocatalytic amplification of amyloid fibrils by secondary nucleation. GO/CFs interfere with secondary nucleation by binding to amyloid fibrils surfaces and blocking the sites that catalyze nucleation.

Figure 4 shows an updated Scheme of oligomer and amyloid fibril formation. GO/CFs are an alternative (off‐pathway), metastable assembly type and form rapidly and extensively above the COC. GO/CFs inhibit amyloid formation by competing for the monomers that are required for amyloid fibril nucleation and elongation.^[11]^ In addition, as we show here, GO/CFs actively inhibit the autocatalytic amplification of fibrils by blocking secondary nucleation sites on amyloid fibrils.

Recently, protofibril–fibril interactions were observed under conditions of biphasic Aβ42 assembly, and the protofibrils were interpreted to represent nuclei formed by secondary nucleation.^[31]^ This interpretation is in conflict with the off‐pathway nature of protofibrils.[^11^, ^13^] The results reported here show that protofibril–fibril interactions do not represent, but rather interfere with secondary nucleation.

The interplay between gO/CFs and amyloid fibrils has a high relevance for AD pathogenesis: GO/CFs, which are thought to represent the main toxic Aβ species,[^8^, ^13^, ^21^, ^32^] were shown to associate with amyloid fibril plaques in vivo, with potential consequences for the neurotoxic activities of both assembly types.[^22^, ^23^] For example, amyloid fibril plaques might serve as reservoir of toxic gO/CFs.[^22^, ^23^] Our results demonstrate that the interaction of gO/CFs with amyloid fibrils affects the kinetics of formation and depletion of the two species. By binding to amyloid fibrils, gO/CFs inhibit formation of new fibrils and thereby delay their own replacement by amyloid fibrils. The dimAβ‐Aβ40 system may serve as a valuable tool for further elucidation of the interplay between gO/CFs and amyloid fibrils.

## Conflict of interest

The authors declare no conflict of interest.

## Supporting information

As a service to our authors and readers, this journal provides supporting information supplied by the authors. Such materials are peer reviewed and may be re‐organized for online delivery, but are not copy‐edited or typeset. Technical support issues arising from supporting information (other than missing files) should be addressed to the authors.

Supplementary
