# Supplementary material for: Protofibril–Fibril Interactions Inhibit Amyloid Fibril Assembly by Obstructing Secondary Nucleation
Source: Angew Chem Int Ed Engl. 2020 Dec 11;60(6):3016–21. doi: 10.1002/anie.202010098 (PMC7898819; doi:10.1002/anie.202010098)
Supplement: Supplementary file 1 — Supplementary [file ANIE-60-3016-s001.pdf]

Angewandte Chemie
Eine Zeitschrift der Gesellschaft Deutscher Chemiker

Supporting Information

Protofibril–Fibril Interactions Inhibit Amyloid Fibril Assembly by Obstructing Secondary Nucleation

Filip Hasecke+, Chamani Niyangoda+, Gustavo Borjas, Jianjun Pan, Garrett Matthews, Martin Muschol, and Wolfgang Hoyer**

anie_202010098_sm_miscellaneous_information.pdf

Table of contents

1. Supporting Experimental Section

2. Supporting Figures

3. Supporting References

1. Supporting Experimental Section

Proteins and chemicals

DimAβ was recombinantly produced as previously described.[1] Aβ40 and Aβ42 were obtained from Bachem or rPeptide. Two-times crystallized and lyophyilized hen egg-white lysozyme (hewL) was obtained from Worthington Biochemical Corporation and used without further purification. Ferritin was obtained from Cytiva.

Preparation of Aβ40

Before use, Aβ40 peptide from Bachem was further purified. The lyophilized powder was reconstituted in 6M guanidinium chloride, 50 mM sodium-phosphate buffer, pH 7.4. Reverse phase high-performance liquid chromatography (RP-HPLC) was performed to remove residual impurities. The sample was loaded onto a semi-preparative Zorbax 300SB-C8 RP-HPLC column (9.4 mm × 250 mm, Agilent) connected to an Agilent 1260 Infinity system with UV detection at 214 nm. Monomeric Aβ40 was eluted in a gradient from 30% (v/v) to 36% acetonitrile in water, 0.1% (v/v) trifluoroacetic acid at 80 °C. Aβ40 containing fractions were pooled, lyophilized, dissolved in HFIP, aliquoted in 1 mg portions, lyophilized again, and stored at RT.

Preparation of monomeric Aβ species for ThT kinetics experiments

For aggregation kinetics experiments, the lyophilized protein (Aβ40 or dimAβ) was reconstituted in 6M guanidinium chloride, 50 mM sodium-phosphate buffer, pH 7.4, and incubated at room temperature for 30 min. The lyophilized powder from rPeptide (Aβ40 or Aβ42) was dissolved directly in 100 mM NaOH at pH 12. For all Aβ peptides, SEC was performed using a Superdex 75 increase column (GE Healthcare) equilibrated with 35 mM Na_2HPO_4 50 mM NaCl, 5 mM NaOH, pH 11. The concentration of the monomeric peptides was measured via UV absorption at 280 nm using the extinction coefficient of 1,490 M^{-1} cm^{-1} for Aβ40 and Aβ42, and 2,980 M^{-1} cm^{-1} for dimAβ. Solutions were kept on ice during subsequent sample preparation. Immediately before the start of ThT kinetics experiments, 1.5% 1 M NaH_2PO_4 was added, yielding 50 mM Na-phosphate, 50 mM NaCl, pH 7.4, as final buffer composition.

Preparation of dimAβ gO/CFs for kinetic assays

DimAβ lyophilisate was resuspended in a small volume (3-5 µl) 50 mM NaOH until completely dissolved. Next, 50 mM Na-phosphate buffer, 50 mM NaCl, pH 7.4, and 50 mM HCl (3-5 µl) were added and immediately mixed, obtaining a final concentration of 10 µM dimAβ. To induce gO/CF formation, dimAβ was incubated at 37°C for 16 – 24 hours.

Preparation of Aβ40 fibril seeds for kinetic assays

Aβ40 lyophilisate was resuspended in a small volume (3-5 µl) 50 mM NaOH until completely dissolved. Next, 50 mM Na-phosphate buffer, 50 mM NaCl, pH, 7.4, 10 µM ThT, and 50 mM HCl (3-5 µl) were added and immediately mixed, obtaining a final concentration of 10 µM Aβ40. Aβ amyloid growth was monitored using a BMG ClarioStar plate reader in 96-well low-binding plates (Greiner). After 16 – 24 hours samples with a steady plateau were taken and combined. Samples were sonicated using a Bandelin Sonopuls utilizing an MS72 sonicator tip. Sonication was performed in 3 pulses of one second sonication and five seconds waiting in between at 25% amplitude strength.

Preparation of isolated hewL fibrils and gO/CFs for interaction experiments

HewL fibrils and gO/CFs were grown and isolated following protocols previously described.[1] In short, lyophilized hewL was dissolved at 14 mM in 25 mM KH_2PO4 buffer with either 50 mM NaCl (fibrils) or 250 mM NaCl (gO/CFs) and incubated for 90 hours (fibrils) or 5 hours (gO/CFs), respectively. RFs and go/CFs were isolated from the residual monomeric background three repeated centrifugation (15,000 rpm for 12 hours, each) with the resulting pellet re-suspended in fresh buffer solution after each round.

Amyloid formation assays using Thioflavin T (ThT) fluorescence

ThT stock solutions were prepared by dissolving 2 mM dye in distilled water and then filtering through a 220 nm syringe filter. Final ThT concentrations were obtained from absorption at a wavelength of 412 nm (ε_{412} = 32,000 M^{-1} cm^{-1}). Aβ amyloid growth kinetics measurements were performed using either a BMG ClarioStar or a BMG Fluostar Optima plate reader with ThT excitation at 445 nm and emission collected at 482 nm in 96-well low-binding half-area plates (Greiner) which were sealed with transparent polypropylene films. Samples contained Aβ species as indicated in the Results and 10 µM ThT in 50 mM Na-phosphate buffer, 50 mM NaCl, pH7.4. Typically, three identical 100 µL samples were incubated at 37 °C. Measurements were taken every 3 minutes without shaking in between.

Data analysis of ThT kinetics

ThT data in Figure 1 was analyzed as described in ref.[1]. Briefly, sigmoidal amyloid growth kinetics below the COC were fit to analytical approximations of nucleated polymerization with secondary nucleation mechanisms. Biphasic growth kinetics above the COC were analyzed in two steps. First, the portion dominated by gO/CF formation was fit to a one-step oligomerization n M → M_n function. To be able to fit the fibril nucleation-growth dominated second part of the kinetics, the oligomerization fit was substracted from the raw data followed by a fit to analytical approximations of nucleated polymerization with secondary nucleation mechanisms as

described before. We defined the RF lag time as the point at which the amplitude of the RF portion of the ThT signal increases beyond a fixed threshold.

Primary and secondary nucleation and elongation models using AmyloFit

Data as shown in Figure 3 E and F was analyzed using the online software AmyloFit.[2] The ThT raw data was uploaded into the fitting software and the triplicates were grouped. Sample and seed concentrations were assigned to the individual groups. The seed particle concentration was defined assuming fibril seed sizes of 1,000 Aβ subunits per seed. The models "Nucleation Elongation" and "Secondary Nucleation Dominated" were used to analyze the data. The variables n_c and n_2 were kept at 2. Only one of the variables k_n, k_+ and k_2 was fitted individually while the others were fitted globally as indicated in the Results.

Secondary nucleation-growth model including binding of gO/CFs to amyloid fibril surface

The effect of gO/CFs on fibril growth was modeled using numerical simulations. The change of fibril mass concentration (M) and fibril particle concentration (P) was calculated over time in one thousand time increments over the observed timespan. The rates for primary nucleation (k_n), elongation (k_+) and secondary nucleation (k_2) for Aβ40 in the presence of 0.1% Aβ40 fibril seeds and in the absence of gO/CFs were obtained from analysis with AmyloFit.

Each time increment included the calculation of the following derivatives:

$\frac{\mathrm{d}P}{\mathrm{d}t} = k_\mathrm{n} \cdot m(t)^{n_\mathrm{c}}$ | Change of fibril particle concentration due to primary nucleation-growth

$\frac{\mathrm{d}M}{\mathrm{d}t} = 2 \cdot k_+ P(t) m(t)$ | Change of fibril mass concentration due to fibril elongation

In our model of secondary nucleation inhibition in response to gO/CF binding to secondary nucleation sites, the formula for secondary nucleation was modified to include the reduction of available fibril surface for secondary nucleation:

$\frac{\mathrm{d}P}{\mathrm{d}t} = k_2 \cdot \left(M(t) - M_\mathrm{blocked}(t)\right) \cdot m(t)^{n_\mathrm{c}}$ | Secondary nucleation including surface inhibition

The blocked fraction was calculated taking the inhibitor concentration [gO/CFs] and the dissociation constant (K_D) into account:

$$K_\mathrm{D} = \frac{[\mathrm{gO/CFs_{free}}][\mathrm{M_{free}}]}{[\mathrm{M_{blocked}}]}$$

$$[\mathrm{M}]_\mathrm{blocked}(t) = \frac{([\mathrm{M}]_\mathrm{total}(t) + [\mathrm{gO/CFs}]_\mathrm{total} + K_\mathrm{D}) - \sqrt{([\mathrm{M}]_\mathrm{total}(t) + [\mathrm{gO/CFs}]_\mathrm{total} + K_\mathrm{D})^2 - 4[\mathrm{M}]_\mathrm{total}(t)[\mathrm{gO/CFs}]_\mathrm{total}}}{2}$$

Atomic force microscopy

For imaging of Aβ assemblies, 25 µl of the samples were applied onto freshly cleaved muscovite mica. After 1 minute of incubation at room temperature, samples were washed three time with 100 µl ddH_2O, and subsequently dried under N_2 gas stream. Imaging was performed

in intermittent contact mode (AC mode) in a JPK Nano Wizard 3 atomic force microscope (JPK, Berlin) using a silicon cantilever with silicon tip (OMCLAC160TS-R3, Olympus) with a typical tip radius of 9 ± 2 nm, a force constant of 26 N/m and resonance frequency around 250 kHz. The images were processed using JPK DP Data Processing Software (version spm-5.0.84) or Gwyddion (version 2.52). For the presented height profiles a polynomial fit was subtracted from each scan line first independently and then using limited data range. False colour height images were superimposed over either amplitude or phase images using Gimp – GNU Image Manipulation Program.

For hewL samples, each of isolated RFs and gO/CFs were brought to 450 mM NaCl, mixed and further diluted to a final concentration ratio of 30:300 μM. 16 μL of this mixture was deposited on freshly cleaved mica, either immediately after mixing or following 15 min of incubation at the amyloid growth temperature of 52 °C. After 5 minute incubation, samples were washed with dH_2O and dried with N_2 gas. AFM imaged were acquired on an MPF-3D (Asylum Research) in taping mode using PFP-FMR-50 silicon tips (Nanosensor) with nominal tip radii of 7 nm. The cantilever had a typical spring constant and resonance frequency of 2 nN/nm and 70 kHz, respectively. It was driven at 60−70 kHz in alternating current mode and at a scan rate of 0.25-0.5 Hz. Images were acquired at 512 × 512 pixel resolution. Amplitude, phase, and height images were collected and processed using the built-in acquisition software.

2. Supporting Figures

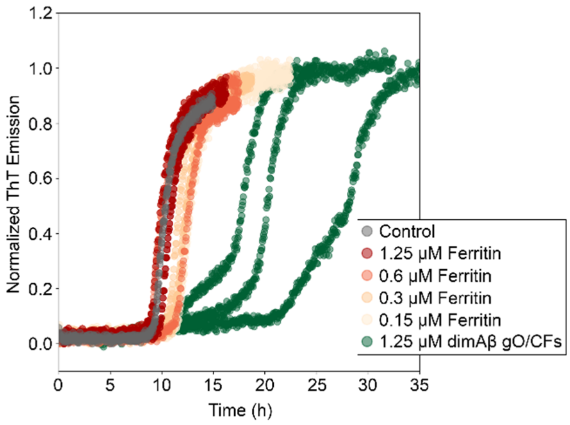

Figure S1: Inhibition of secondary nucleation is not a universal feature of polypeptide assemblies in the size range of gO/CFs. Secondary nucleation-elongation of Aβ40 fibril seeds by Aβ40 monomers in the absence (grey) or presence of either dimAβ gO/CFs (green) or the 440 kD-protein ferritin (red), which is a 24-mer of helical bundles.

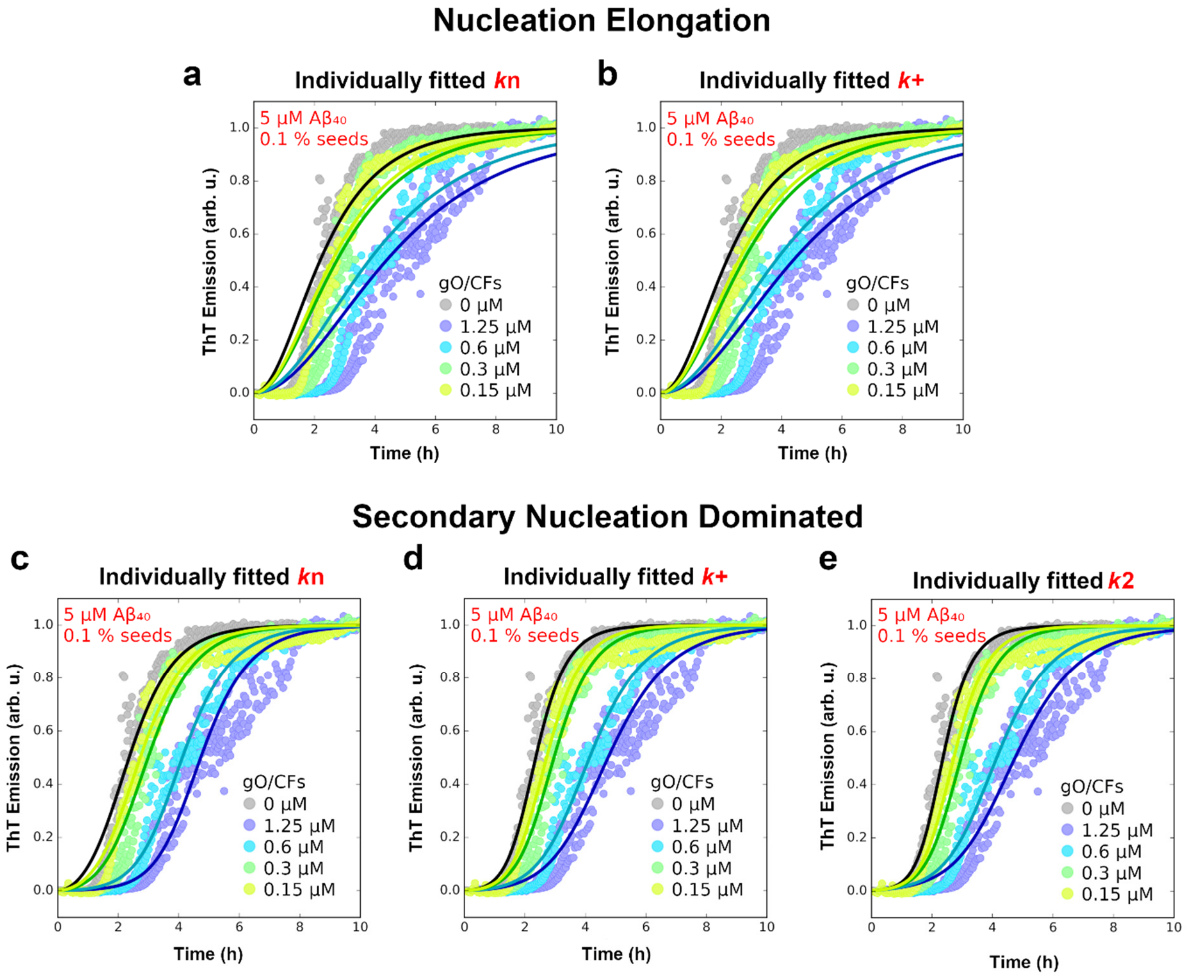

Figure S2: Comparison of the “Nucleation Elongation” and “Secondary Nucleation Dominated” models of AmyloFit. ThT traces from 5 µM Aβ40 seeded with 0.1 % Aβ40 seeds treated with inrcreasing concentrations of dimAβ gO/CFs were analyzed. The effect of gO/CFs on the kinetic rates of fibril nucleation and growth was analyzed by fitting one of the kinetic rates k_n, k_+, or k_2 individually while the other rates were fitted globally. a), b) Fits to the “Nucleation Elongation” model, either fitting k_n (a) or k_+ (b) individually. c)-e) Fits to the “Secondary Nucleation Dominated” model, either fitting k_n (c), k_+ (d), or k_2 (e) individually.

3. Supporting References

[1] F. Hasecke, T. Miti, C. Perez, J. Barton, D. Scholzel, L. Gremer, C. S. R. Gruning, G. Matthews, G. Meisl, T. P. J. Knowles, et al., *Chem. Sci.* **2018**, *9*, 5937-5948.

[2] G. Meisl, J. B. Kirkegaard, P. Arosio, T. C. Michaels, M. Vendruscolo, C. M. Dobson, S. Linse, T. P. Knowles, *Nat. Protoc.* **2016**, *11*, 252-272.
